# Hyaluronic Acid Viscosupplement Modulates Inflammatory Mediators in Chondrocyte and Macrophage Coculture via MAPK and NF-κB Signaling Pathways

**DOI:** 10.1021/acsomega.4c01911

**Published:** 2024-05-01

**Authors:** Sree Samanvitha Kuppa, Ju Yeon Kang, Hong Yeol Yang, Seok Cheol Lee, Jaishree Sankaranarayanan, Hyung Keun Kim, Jong Keun Seon

**Affiliations:** † Department of Biomedical Sciences, Chonnam National University Medical School, Hwasun 58128, Korea; ‡ Department of Orthopaedics Surgery, Center for Joint Disease of Chonnam National University Hwasun Hospital, 322 Seoyang-ro, Hwasun-eup, Jeonnam 519-763, Korea; § Korea Biomedical Materials and Devices Innovation Research Center of Chonnam National University Hospital, 42, Jebong-ro, Dong-gu, Gwangju 501-757, Korea

## Abstract

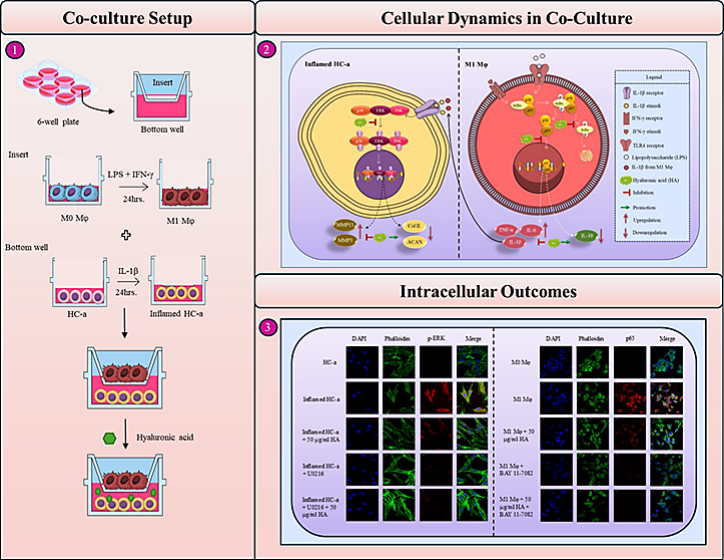

Osteoarthritis (OA) is a chronic musculoskeletal disorder characterized by cartilage degeneration and synovial inflammation. Paracrine interactions between chondrocytes and macrophages play an essential role in the onset and progression of OA. In this study, in replicating the inflammatory response during OA pathogenesis, chondrocytes were treated with interleukin-1β (IL-1β), and macrophages were treated with lipopolysaccharide and interferon-γ. In addition, a coculture system was developed to simulate the biological situation in the joint. In this study, we examined the impact of hyaluronic acid (HA) viscosupplement, particularly Hyruan Plus, on chondrocytes and macrophages. Notably, this viscosupplement has demonstrated promising outcomes in reducing inflammation; however, the underlying mechanism of action remains elusive. The viscosupplement attenuated inflammation, showing an inhibitory effect on nitric oxide production, downregulating proinflammatory cytokines such as matrix metalloproteinases (MMP13 and MMP3), and upregulating the expression levels of type II collagen and aggrecan in chondrocytes. HA also reduced the expression level of inflammatory cytokines such as IL-1β, TNF-α, and IL-6 in macrophages, and HA exerted an overall protective effect by partially suppressing the MAPK pathway in chondrocytes and p65/NF-κB signaling in macrophages. Therefore, HA shows potential as a viscosupplement for treating arthritic joints.

## Introduction

Osteoarthritis (OA) is a common degenerative joint disease characterized by the breakdown of cartilage in the joints. It causes symptoms such as joint pain, stiffness, swelling, and reduced range of motion.^1^ The risk factors for OA include aging, joint injury, obesity, and genetic predisposition.^2^ Inflammation plays a significant role in cartilage breakdown and synovitis in patients with OA. It is triggered by proinflammatory molecules released from damaged cartilage, which activate immune cells such as macrophages (Mφ). These immune cells release additional inflammatory molecules and enzymes, such as matrix metalloproteinases (MMPs), which degrade cartilage components.^3^ These mediators attract immune cells, degrade the extracellular matrix, promote inflammation, and contribute to tissue damage. Mφ can also indirectly affect chondrocytes by inducing the production of inflammatory molecules that disrupt cartilage balance, stimulate chondrocyte apoptosis, and alter chondrocyte metabolism.^4^ Inflammatory M1Mφ can negatively affect the chondrogenesis of mesenchymal stem cells. M1Mφ can also modulate the function of synovial fibroblasts and chondrocytes, leading to increased inflammation and increased production of inflammatory molecules.^5^ Furthermore, chondrocytes and Mφ engage in reciprocal interactions, thereby altering the expression profiles of degradative enzymes and their inhibitors, ultimately leading to cartilage degradation.^4^ By contrast, M2Mφ plays a critical role in tissue repair, and it can contribute to the restoration of damaged articular cartilage.^6^ Coculture models incorporating chondrocytes and Mφ have been widely used to investigate the impact of inflammation and paracrine interactions among these cell types. In a notable study by Dreier et al. a coculture approach was used to investigate the interplay between chondrocytes and monocytes/Mφ. Using this method, chondrocytes were found to exert a significant influence on the expression and activation of pro-MMP-9 (gelatinase B) in Mφ.^7^ Similarly, Bauer et al. used a coculture system comprising proinflammatory Mφ and chondrocytes from individuals with OA. Their findings revealed an upregulation in the expression level of MMPs and proinflammatory cytokines, which is indicative of the early stages of OA. This study also demonstrated the ability of high-molecular-weight hyaluronic acid (HMWHA) to protect chondrocytes against the inflammatory response.^8^ Collectively, these findings indicate the intricate communication and regulatory mechanisms that can be unveiled through coculture systems.

These observations are relevant when considering the current treatment options for OA, which include various treatment strategies. The current treatment options for OA include nonpharmacological strategies, such as weight management, physical therapy, and assistive devices, as well as pharmacological interventions, including analgesics, topical treatments, and corticosteroid injections.^9^ These traditional treatments provide temporary relief without addressing the root cause of OA, whereas surgical interventions, such as joint replacements, involve risks and prolonged recovery periods. By contrast, viscosupplements such as hyaluronic acid (HA) injections provide a promising alternative by delivering enduring pain relief and potentially slowing OA progression.^10^ HA, a naturally occurring high-molecular-weight molecule in cartilage and synovial fluid, undergoes depolymerization during OA progression, reducing the mechanical properties of the synovial fluid. Exogenous HA injections alleviate this deficiency through proteoglycan synthesis and anti-inflammatory effects.^11^ Viscosupplements serve as lubricants and shock absorbers in the joint, providing minimally invasive, targeted relief and an opioid-sparing option. They can also promote cartilage preservation and improve joint function, often complementing other therapies such as physical therapy.^12,13^ Nonetheless, their effectiveness varies among individuals and multiple injections may be necessary.

This research study aims to investigate the mechanism of action underlying the effectiveness of sodium hyaluronate, specifically Hyruan Plus (LG Chemical Ltd., Iksan), on human articular chondrocytes (HC-a) and THP-1-derived Mφ. Hyruan Plus is a commonly used viscosupplement for the management of OA; however, its precise mechanism of action remains elusive. Therefore, our study was designed to investigate and shed light on this mechanism. Our investigation yielded encouraging findings regarding its ability to mitigate inflammation. In exploring the application potential of viscosupplements, we established a coculture system combining chondrocytes (HC-a) and THP-1-derived Mφ, which provides an advantageous platform for investigating various aspects of the OA joint. By incorporating proinflammatory stimuli such as interleukin-1 beta (IL-1β), lipopolysaccharide (LPS), and interferon-gamma (IFN-γ), we simulated the inflammatory conditions observed in OA. This coculture system aimed to elucidate the paracrine interactions between HC-a and THP-1 cells and to examine the in vitro production of various proinflammatory cytokines, namely, MMP3, MMP13, IL-1β, TNF-α, and IL-6, within the HC-a/THP-1-derived Mφ coculture model, which, to our knowledge, is the first of its kind to investigate the role of viscosupplements in this context. In addition, we aimed to evaluate the ability of the viscosupplement to mitigate inflammation in this model by examining its impact on MAPK and NF-κB signaling pathways. The findings of this study demonstrated that the viscosupplement effectively inhibited the activation of the MAPK and NF-κB signaling pathways, thereby reducing inflammation.

## Methods

### Reagents

Dulbecco’s Modified Eagle’s Medium (DMEM), fetal bovine serum (FBS), and penicillin–streptomycin antibiotics (5000 U/mL) were procured from Gibco (Life Technologies, Thermofisher, USA). Roswell Park Memorial Institute medium (RPMI 1640) supplemented with l-glutamine and sodium bicarbonate was purchased from WELGENE (Korea). Hyruan Plus, a linear HMWHA with a mean molecular weight of 3000 kDa, was obtained from LG Life Sciences (Iksan, South Korea) and further diluted in PBS WELGENE (Korea). IL-1β was purchased from R&D Systems (Minneapolis, MN, USA). Phorbol 12-myristate 13-acetate (PMA) was purchased from Sigma-Aldrich (USA). LPS and IFN-γ were purchased from Merck Millipore (Darmstadt, Germany). RNAiso plus was acquired from TaKaRa (Dalian, China). The BCA kit was obtained from Pierce (Thermo Scientific, USA). Antibodies against type II collagen (Col II, Abcam, USA), aggrecan (ACAN, Abcam, USA), matrix metalloproteinase 3 (MMP3, BioLegend, USA), matrix metalloproteinase 13 (MMP13, Bioss, USA), IL-1β (Santa Cruz, USA), tumor necrosis factor alpha (TNF-α, Abcam, USA), IL-6 (Proteintech, USA), IL-10 (AbClonal, USA), p65 (Cell Signaling, USA), phosphorylated p65 (p-p65, Cell Signaling, USA), IκBα (Cell Signaling, USA), phosphorylated IκBα (p-IκBα, Cell Signaling, USA), P44/42 MAPK (ERK1/2) antibody (Cell Signaling, USA), phosphorylated P44/42 MAPK (ERK1/2, Thr202/Tyr204) antibody (Cell Signaling, USA), SAPK/JNK (Cell Signaling, USA), and phosphorylated SAPK/JNK (Thr183/Tyr185, Cell Signaling, USA) were purchased. In addition, we obtained conjugated goat antirabbit secondary antibody (H+L, Novex Life Technologies, Thermo Fisher Scientific, USA) and goat antimouse IgG (ZyMax, Thermo Fisher Scientific, USA). The chemiluminescence reagent was purchased from Amersham Biosciences (UK).

### Cell Culture of HC-a and Differentiation of THP-1 Monocytes to M0Mφ

The HC-a cell line was purchased from ScienCell (#4650, Carlsbad, USA)^14,15^ and seeded at a density of 1 × 10^6^ cells/mL in DMEM supplemented with 10% FBS and 1% penicillin/streptomycin. Cells were cultured at 37 °C in a 5% CO_2_ environment. For all experiments, passages 2–3 HC-a were used within 1 week after seeding.

Human monocytic THP-1 (ATCC TIB-202) cells were maintained in an RPMI 1640 culture medium supplemented with 10% FBS and 1% penicillin/streptomycin. A transition from RPMI to DMEM culture media was performed to ensure compatibility during coculture experiments.^16,17^ THP-1 cells were thawed in 10% DMEM to assess their viability. Acclimation allowed the cells to quickly adapt to the new medium. Subsequently, a gradual switch from RPMI to DMEM media was implemented over several passages, enabling the cells to gradually acclimate to the new medium (Supplementary Figure 1 demonstrates the absence of alterations in morphological characteristics and gene expression).

**Figure 1:**
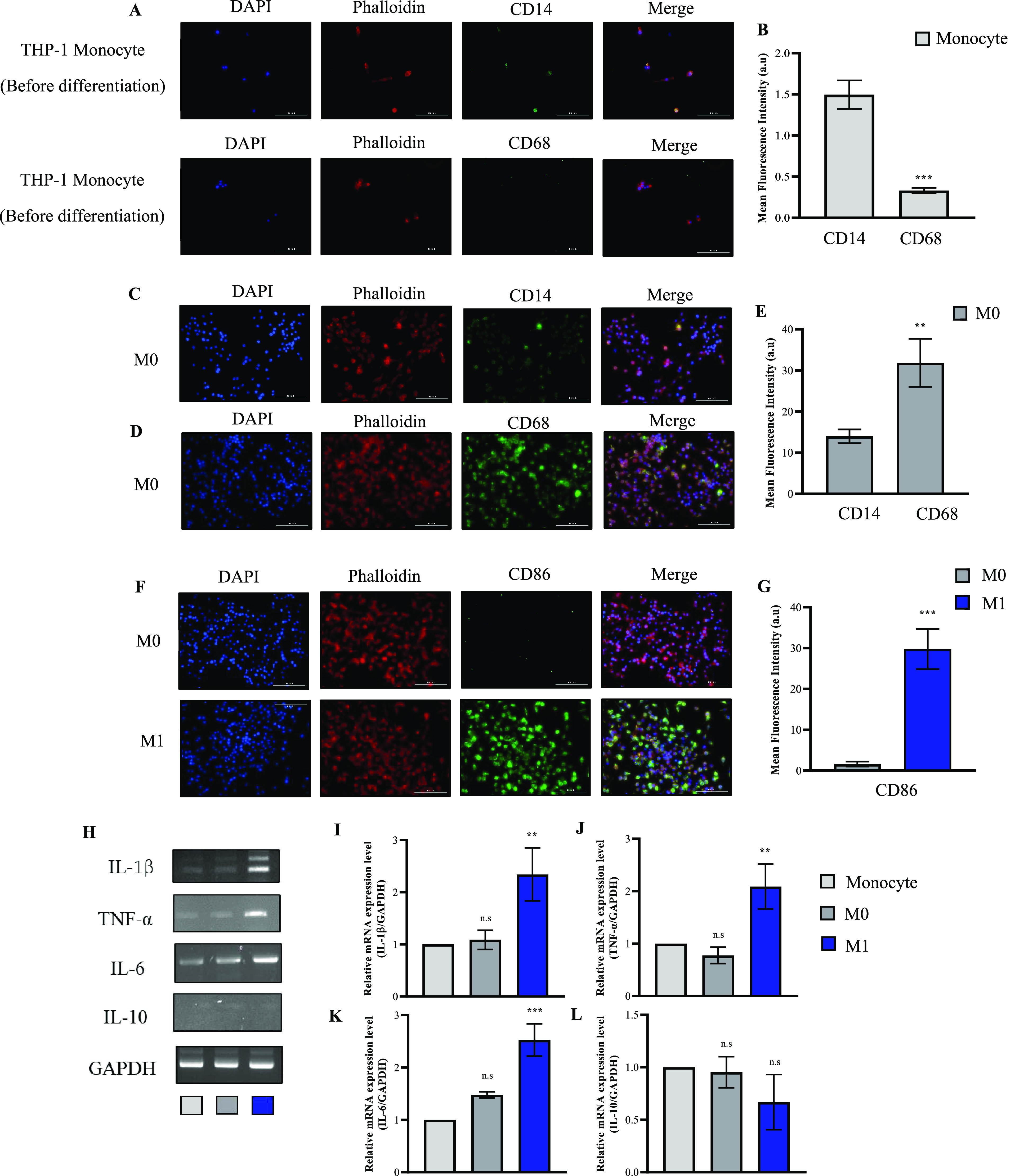
Immunofluorescence staining of undifferentiated and differentiated THP-1 cells. (A) Representative immunofluorescence image of THP-1 cells that were stained with CD14 and CD68; (B) quantification of monocytes containing positively stained cells of CD14 and CD68 (***p < 0.001 in comparison to THP-1 cells stained with CD14). (C) Immunofluorescence image of differentiated THP-1 cells (M0Mφ) that were stained with CD14 and (D) CD68-stained cells in M0Mφ. (E) Quantification of M0Mφ containing positively stained cells of CD14 and CD68 (**p < 0.01 relative to M0Mφ that were stained with CD14). (F) Illustrative immunofluorescence image of M0Mφ, and M1Mφ cells that were stained with CD86; (G) quantification of positively stained CD86 cells in differentiated M0Mφ, and M1Mφ cells (***p < 0.001 when compared to M0Mφ cells that were not stained with CD86). (H) PCR analysis of IL-1β, TNF-α, IL-6, and IL-10 gene expression in monocytes M0 and M1 cells, and (I–L) quantification of gene expression (n.s.: no significance; * *p* < 0.05, ** *p* < 0.01 and *** *p* < 0.001 in comparison to monocytes).

In activating monocytes and facilitating their differentiation into Mφ, the approach outlined by Shiratori et al. and Michiels et al. was adopted, as it has been shown to yield a phenotype suitable for inflammation-related cell culture studies.^18,19^ THP-1 cells were cultured in a 100 mm culture dish at a density of 1 × 10^6^ cells/mL. PMA was introduced into the culture medium at a concentration of 50 ng/mL. Then, the cells were incubated with PMA for 48 h. After incubation, the PMA-containing medium was substituted with a fresh medium lacking PMA, and the cells were further incubated for an additional 24 h to promote the differentiation of THP-1 monocytes into Mφ.

### Differentiation of M0 to M1Mφ, Activation of HC-a, and Coculture Procedures

The coculture system was established using a six-well 0.4-μm porous cell culture insert (Corning, USA). Initially, THP-1 monocytes were seeded at a concentration of 3 × 10^5^ cells/mL into the upper chamber of the Transwell. The monocytes were treated with 50 ng/mL PMA for 48 h to induce differentiation. Following incubation, the PMA-containing medium was replaced with fresh medium, excluding PMA, and the cells were further incubated for 24 h to facilitate their differentiation into Mφ. Subsequently, Mφ was washed two times with phosphate-buffered saline (PBS) and cultured in media supplemented with 20 ng/mL IFN-γ and 50 ng/mL LPS for 24 h to promote differentiation into M1Mφ (Supplementary Figure 2 shows the cytotoxicity of different concentrations of IFN-γ and LPS).

**Figure 2:**
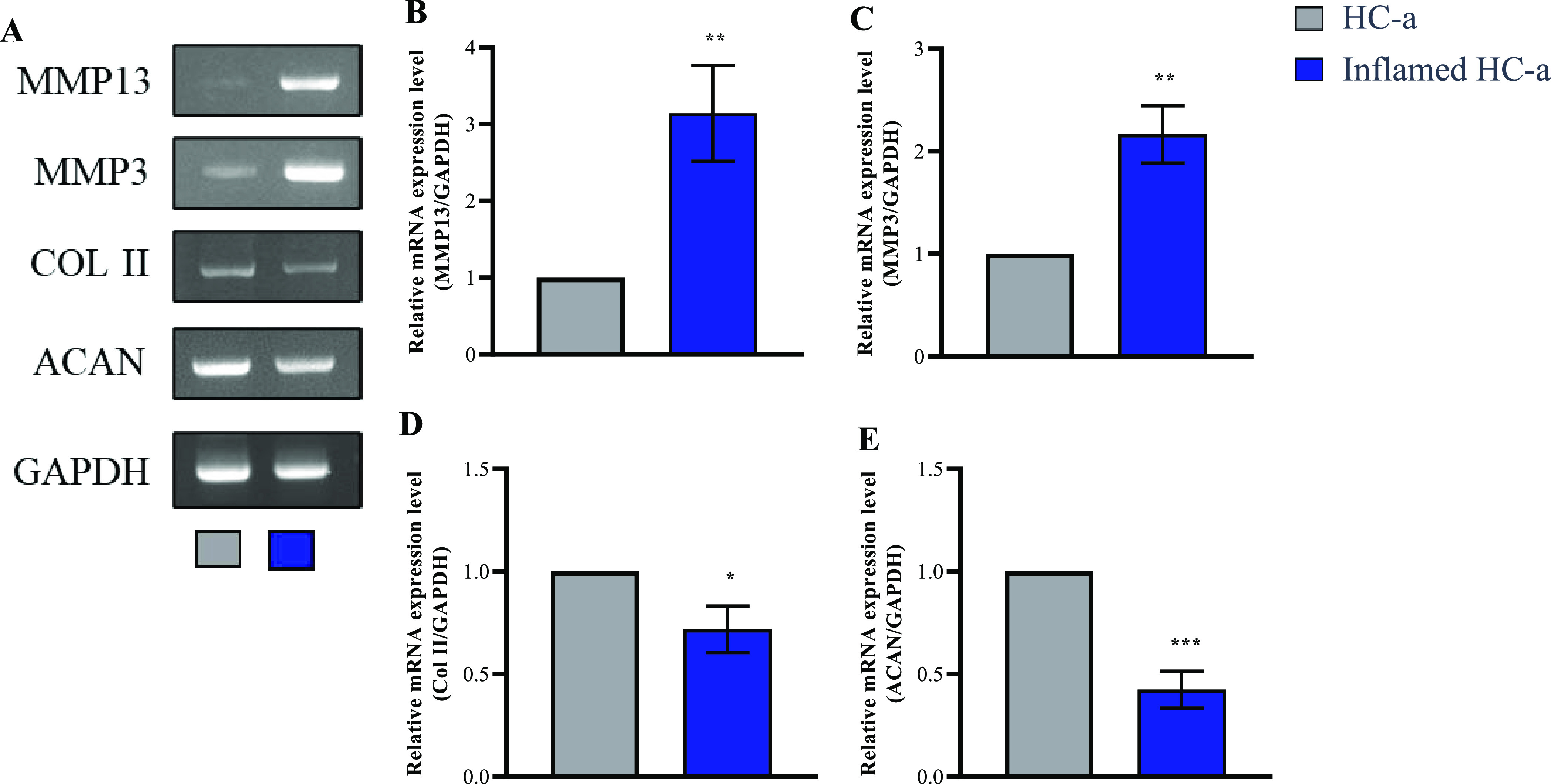
Gene expression analysis of MMP13, MMP3, Col II and ACAN in individually cultured HC-a cells. This figure shows the RT-PCR analysis, focusing on (A) gene expression patterns of MMP13, MMP3, type II collagen (Col II) and aggrecan (ACAN) and (B–E) quantification of these key markers involved in extracellular matrix remodeling (* *p* < 0.05, ** *p* < 0.01, and *** *p* < 0.001 compared to control HC-a).

Simultaneously, before the establishment of the coculture system, HC-a cells were seeded at a concentration of 3 × 10^5^ cells/mL in the lower chamber and allowed to attach for 24 h. Following attachment, the culture medium was supplemented with IL-1β at a concentration of 10 ng/mL and incubated for an additional 24 h to induce inflammation in the HC-a cells.

Subsequently, the chambers housing THP-1-derived M1Mφ were directly overlaid onto six-well plates containing the inflamed HC-a cells, establishing a coculture system. The resulting cells within the coculture system were cultured using DMEM and subsequently incubated in the presence or absence of varying concentrations of HA viscosupplement (5, 50, and 500 μg/mL) for 24 h. Similarly, HC-a cells without IL-1β treatment and THP-1-derived M0Mφ were separately cocultured in six-well plates for 24 h, which served as the appropriate controls.

### Total-RNA Extraction and RT-PCR

Gene expression analysis was conducted on THP-1-derived Mφ and HC-a cells. Total RNA was harvested from six-well plates for each cell type. RNAisoPlus reagent (TaKaRa, Japan) was used to extract RNA. The RNA pellet was resuspended in 20 μL of RNase-free water. For cDNA synthesis, 1 μg of RNA was added to the RT-premix from Bioneer (Korea), following the manufacturer’s instructions. The reaction mixture was incubated at 42 °C for 60 min to enable cDNA synthesis, followed by heat inactivation of the reverse transcriptase at 70 °C for 10 min. The resulting cDNA was diluted with RNase-free water to the desired concentration and added to the PCR master mix from Bioneer (Korea). PCR amplification was performed as follows: initial denaturation step at 95 °C for 5 min, further denaturation at 95 °C for 20 s, an annealing step at 45–65 °C optimized for the respective primers (Table 1) for 20 s, and a polymerization step at 72 °C for 30 s for the target genes.

**Table 1:** Human Primer Sequences Used in This Study

**no.**	**primer name**	**forward primer**	**reverse primer**
1	Human IL-1β	5′-TGCCTTAGGGTAGTGCT-3′	5′-GCGGTTGCTCATCAGA-3′
2	Human TNF-α	5′-AGGCGGTGCTTGTTCCTC-3′	5′-GTTCGAGAAGATGATCTGACTGCC-3′
3	Human IL-6	5′-GGATGCTTCCAATCTGGATTCAATGAG-3′	5′-CGCAGAATGAGATGAGTTGTCATGTCC-3′
4	Human IL-10	5′-AACCTGCCTAACATGCTTCG-3′,	5′-GGGAAGAAATCGATGACAGC-3′
5	Human MMP3	5′-GGCAGTTTGCTCAGCCTATC-3′	5′-GTCACCTCCAATCCAAGGAA-3′
6	Human MMP13	5′-GATGAAGACCCCAACCCTAAA-3′	5′-CTGGCCAAAATGATTTCGTTA-3′
7	Human Col II	5′-TCTGCAACATGGAGACTGGC-3′	5′-GAAGCAGACCGGCCCTATGT-3′
8	Human ACAN	5′-ACGAGTGGCAGCGGTGAAT-3′	5′-GCCCTTCTCCTGCCTCTTG-3′
9	Human GAPDH	5′-ACCACAGTCCATGCCATCAC-3′	5′-TCCACCACCCTGTTGCTGTA-3′

### Protein Extraction and Western Blot

Cells were collected from the coculture system and washed with ice-cold PBS. Proteins from the cell lysate were extracted using ice-cold radioimmunoprecipitation assay buffer supplemented with protease and phosphatase inhibitors. The protein samples were denatured by heating at 95 °C for 5 min. The denatured proteins were loaded onto a polyacrylamide gel and subjected to electrophoresis to separate them by size. Subsequently, the separated proteins were transferred from the gel to a polyvinylidene fluoride membrane. Then, the membrane was blocked with 5% bovine serum albumin (BSA) in Tris-buffered saline with Tween 20 (TBST) for 1 h at room temperature (RT) to prevent nonspecific binding. Next, the membrane was incubated overnight at 4 °C with primary antibodies such as MMP3, MMP13, Col II, ACAN, p38, pp38, total JNK, p-JNK, total ERK, p-ERK, IL-1β, TNF-α, IL-6, IL-10, p65, p-p65, IκBα, and p-IκBα specific to the protein of interest. After incubation, the membrane was washed with TBST to remove unbound antibodies and incubated with a secondary antibody conjugated to horseradish peroxidase for 1 h at RT. Following additional washes with TBST, the protein of interest was visualized using an enhanced chemiluminescence substrate. Protein expression levels were analyzed by using ImageJ.

### Inhibitor Study

To understand the underlying mechanism involved in the interaction between HC-a and THP-1-derived Mφ, we used specific inhibitors and assessed the expression of downstream mediators including MMP13 in HC-a cells and IL-1β in Mφ. This study used SB203580 for p38, U0126 for ERK, SP600125 for JNK in the MAPK pathway, and BAY 11-7082 to inhibit NF-κB translocation. All inhibitors were applied at a concentration of 10 μM, as supported by the relevant literature.^20^ The experiment involved the addition of inhibitors to both HC-a cells and Mφ for a period of 24 h. After this time of incubation, the inhibitors were removed when IL-1β was added to generate inflammation in HC-a cells and when Mφ was differentiated using LPS and IFN-γ. Following the differentiation of the cells, the coculture system was incubated for 24 h to allow for the effective action of the inhibitors. Subsequently, cells were harvested from the culture system for subsequent analysis using western blotting.

### Immunofluorescence

Immunofluorescence analysis was performed on a coculture system in the presence and absence of HA to validate and reinforce the findings obtained from western blotting. HC-a cells were cultivated on sterile glass coverslips placed in the bottom well chamber of a six-well culture plate, while Mφ cells were grown in the Transwell system, as previously described. After the designated treatment was completed, the media were aspirated from the well plate and Transwell, and the cells were gently washed three times with ice-cold PBS for 5 min per wash. Subsequently, the cells in the well plate and Transwell membrane were fixed with a fixative solution for 10 min at RT and washed three times with PBS. For the Transwell membrane alone, the treated membranes were carefully cut using a scalpel and transferred to a new six-well plate containing PBS. Following permeabilization of the cells on the coverslip and Transwell membrane using 0.1% Triton X-100 for 5 min at RT, a washing cycle with PBS was performed. Subsequently, the cells were subjected to blocking using 2% BSA for 1 h. Following the blocking step, the cells were incubated overnight at 4 °C on a platform rocker with primary antibodies specific to MMP13 and Col II for HC-a, as well as specific to IL-1β and IL-10 for Mφ. The primary antibodies were appropriately diluted in 1% BSA. On the following day, the cells were washed three times with PBS and then exposed to FITC-tagged secondary antibodies and phalloidin in a dark environment at RT for 2 h. After incubation with antibodies, the slides were washed two times with PBS and subsequently stained with nuclear marker 4′,6-diamidino-2-phenylindole (DAPI) for 5 min to ensure protection from light. Following three additional washes with deionized water, the membranes were transferred onto slides and mounted by using a suitable mounting medium. Staining was visualized using a fluorescence microscope (BioTek Lionheart FX) at a magnification of 20×.

### Confocal Microscopy

We used confocal microscopy using inhibitors such as BAY 11-7082 and U0126 to investigate the intracellular colocalization of p65 in Mφ and p-ERK in HC-a within the cytoplasm or nucleus. Following a protocol similar to the immunofluorescence procedure, the primary antibodies were replaced with antibodies specific to p-ERK and p65. The cellular samples were subsequently examined using a confocal fluorescence microscope (ZEISS LSM 980) at a magnification of 40× to capture the desired images and analyze the colocalization patterns.

### Statistical Analysis

All experimental procedures were conducted independently and replicated three times to ensure statistical robustness. Statistical analysis was performed using GraphPad Prism version 9 (GraphPad Software, San Diego, CA, USA). In determining significant differences, a one-way ANOVA followed by Tukey’s multiple comparison test was used for comparisons among multiple groups, whereas an unpaired Student’s *t* test was used for comparisons between two groups. The significance criteria were denoted as follows: *p* < 0.05 for one asterisk (# and *), *p* < 0.01 for two asterisks (## and **), *p* < 0.001 for three asterisks (### and ***), and *p* < 0.0001 for four asterisks (#### and ****).

## Results

### Differentiated and Activated Mφ

Based on our evaluation of the adherence level and phenotypic traits displayed by the THP-1 derived Mφ population, we chose a concentration of 50 ng/mL PMA. In order to confirm the transformation of THP-1 monocytes into M0 Mφ using a concentration of 50 ng/mL PMA, we performed immunofluorescence analysis to evaluate the presence of the CD68 surface marker, which is recognized for its high expression in Mφ. The obtained results shown in Figure 1A–E demonstrate the absence of CD68 expression in THP-1 monocytes, whereas PMA-differentiated Mφ exhibits strong CD68 expression. CD86 was used as a marker for M1Mφ to determine the activation of M0Mφ toward the M1 phenotype by using 20 ng/mL of IFN-γ and 50 ng/mL of LPS. Figure 1F illustrates the significantly elevated CD86 expression level in the M1Mφ group compared to M0Mφ, providing additional evidence for the activation of M0Mφ into the M1 inflammatory phenotype. In further validating the immunofluorescence findings, RT-PCR analysis was performed to assess the expression levels of proinflammatory markers in M1Mφ compared with M0Mφ and monocytes. The selected proinflammatory markers for analysis were IL-1β, TNF-α, IL-6, and IL-10 (Supplementary Figure 3 is to validate positive controls for IL-10. The positive control was necessary due to the absence of IL-10 bands in Figure 1H. In order to clarify the expression of IL-10, we employed M2Mφ as a positive control, which effectively demonstrated the presence of IL-10). As depicted in Figure 1H, the gene expression level of these proinflammatory markers was considerably higher in M1Mφ than in M0Mφ and monocytes, confirming the activation of M0Mφ into an inflammatory phenotype.

### HC-a Activation

In validating the inflammatory response in HC-a induced by 10 ng/mL IL-1β, the expression level of proinflammatory marker genes was assessed using RT-PCR. The selected markers, MMP3 and MMP13, are well-established indicators of inflammation in HC-a and are associated with cartilage degradation. MMP13 plays a crucial role in cartilage degradation by specifically targeting Col II, whereas MMP3 is involved in cartilage matrix degradation and is upregulated in response to axial compression. As depicted in Figure 2A, the HC-a cells treated with IL-1β exhibited significantly elevated expression levels of MMP3 and MMP13 and suppressed the expression of Col II and ACAN.

### In-Vitro OA Mimicking Coculture Model Establishment

**Figure 3:**
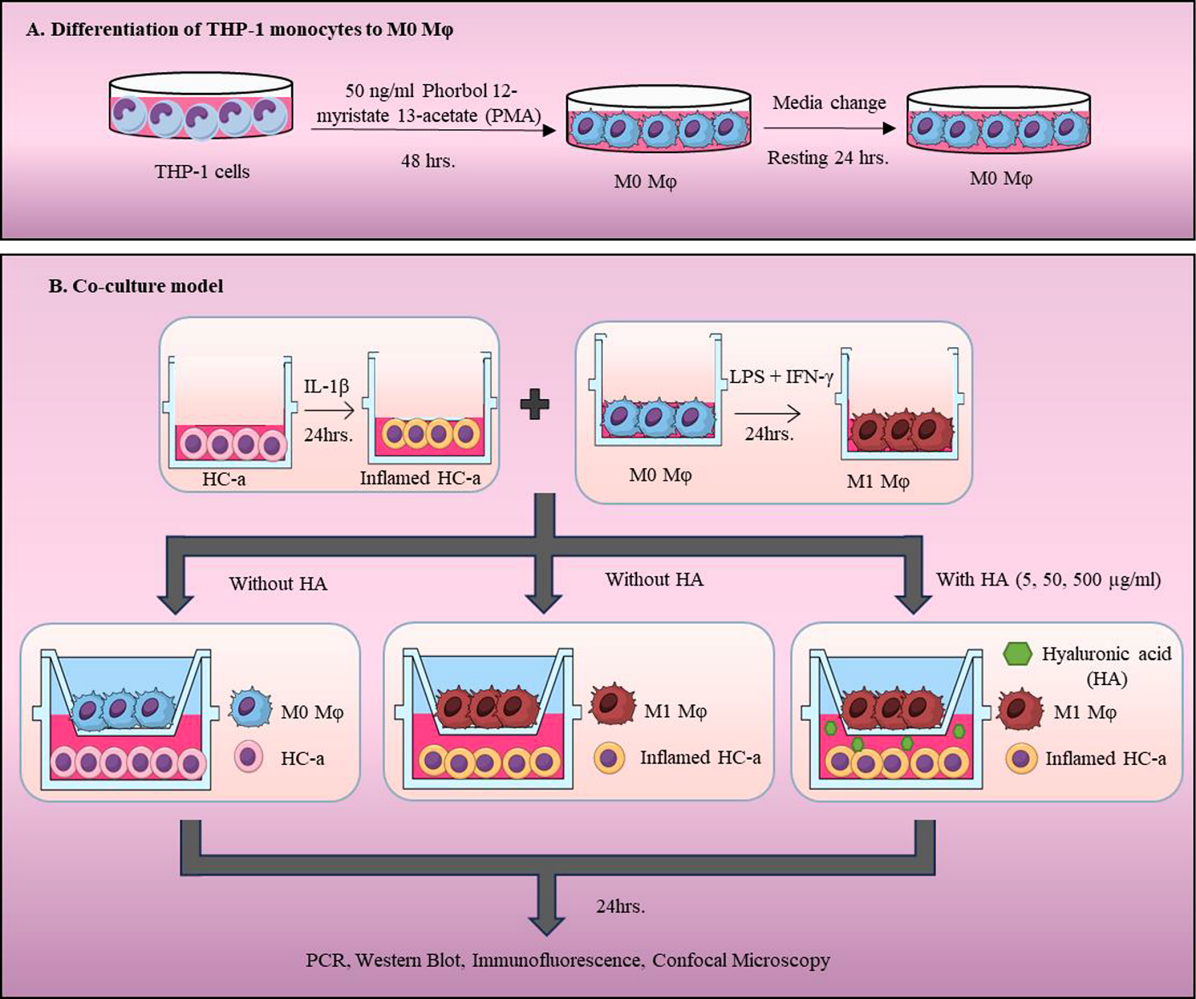
Graphical representation depicting the coculture model employed in the study. The provided figure represents the conceptual framework of the coculture model and the particular cells used for the study. (A) Initial differentiation of monocytes into M0 macrophages, which are thereafter employed in the coculture model. (B) Interaction between HC-a and THP-1 derived Mφ cells in a controlled experimental setting, in both the presence and absence of various concentrations of hyaluronic acid (HA) viscosupplement.

We developed a simple in vitro model of OA to establish a reliable platform for studying the effects of viscosupplements. We used a chondrocyte cell line (HC-a) to ensure consistent conditions. HC-a was treated with IL-1β at a concentration of 10 ng/mL to induce inflammation. Next, we utilized THP-1 cells, a well-established cell line widely used for studying monocyte/Mφ functions, mechanisms, and signaling pathways. We differentiated THP-1 cells into Mφ-like cells using 50 ng/mL PMA and further activated them into an M1 phenotypic state using IFN-γ (20 ng/mL) and LPS (50 ng/mL) (Figure 3A). Subsequently, activated Mφ were incorporated into the coculture system to investigate the impact of the cells in the presence or absence of HA (Figure 3B).

### Validation of Induced Inflammation in the Coculture Model

**Figure 4:**
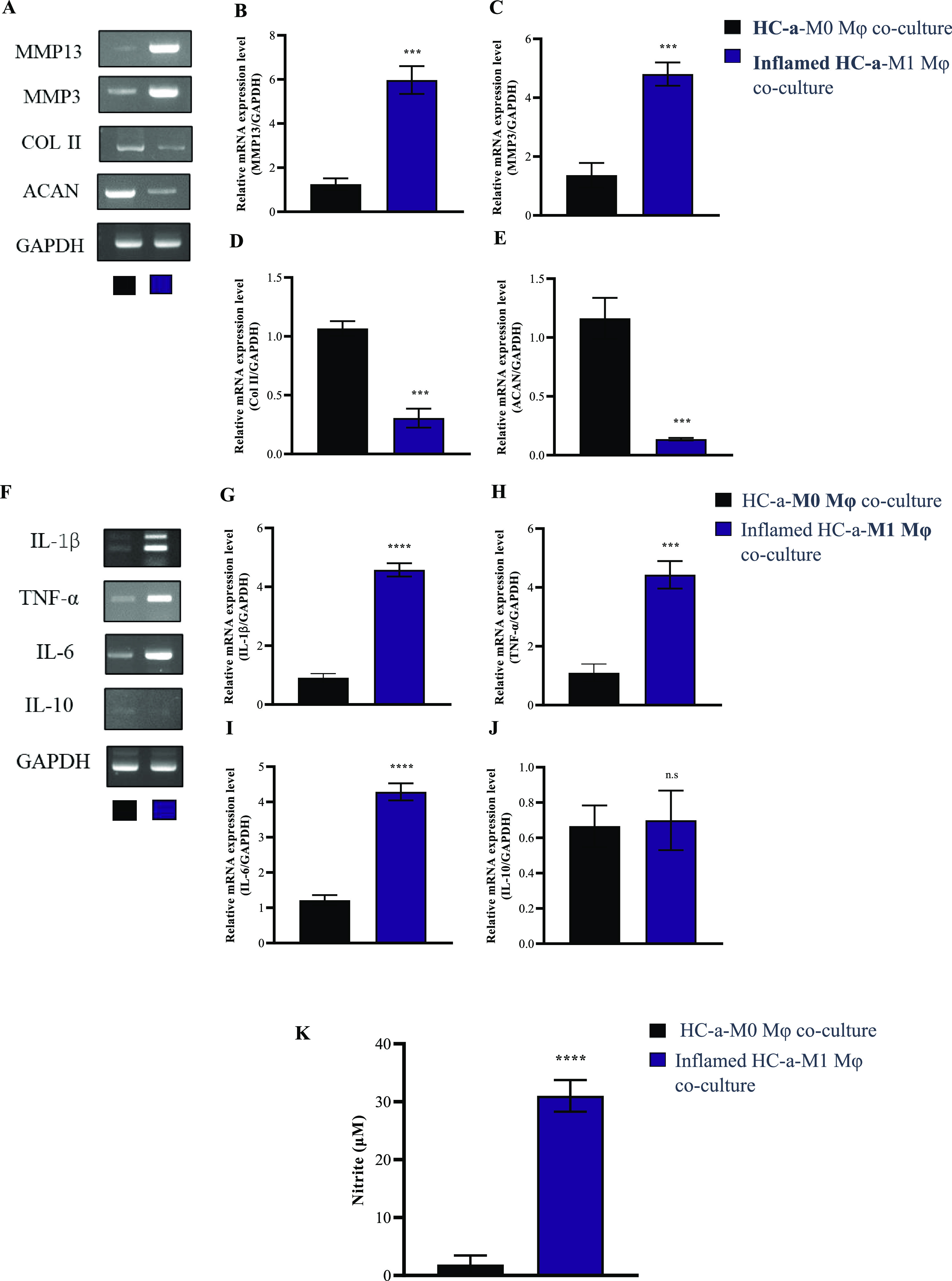
Validation of induced inflammation in the coculture model. (A) Gene expression analysis of cytokines released from cocultured HC-a and inflamed HC-a (MMP13, MMP3, Col II, and ACAN) and (B–E) quantification of the markers (n.s.: no significance; ***p < 0.001 compared to control HC-a cocultured with M0Mφ cells). (F) Gene expression analysis of cytokines released from cocultured M0 and M1Mφ (IL-1β, TNF-α, IL-6, and IL-10) and (G–J) the quantification of the markers (n.s.: no significance; ***p < 0.001, ****p < 0.0001 compared to control M0Mφ cocultured with HC-a cells). (K) Nitric oxide release estimated in coculture of healthy cells and inflamed cells (****p < 0.0001 compared to control coculture of healthy HC-a and M0Mφ cells).

We investigated whether coculturing inflamed HC-a cells with the M1 inflammatory phenotype for 24 h would lead to an inflammatory response in both cell types. Figure 4 illustrates this comparison with a control group in which noninflamed HC-a cells were cocultured with M0Mφ. We evaluated the two coculture models (healthy and inflamed) based on the release of nitric oxide (NO) and proinflammatory cytokines in both cell types. Coculturing inflamed HC-a cells with the inflammatory phenotype (differentiated M1) for 24 h resulted in a significant increase in proinflammatory cytokines in both cell types and NO formation. As shown in Figure 4K the stable coculture of noninflamed HC-a cells and M0Mφ for 24 h did not induce an inflammatory response whereas the inflammatory coculture group showed increased NO production. Supplementary Figure 4 shows NO formation in the presence and absence of various concentrations of (5, 50, and 500 μg/mL) HA.

### Inhibition of Proinflammatory Cytokine Release in Inflamed HC-a Cells in Coculture via HA Treatment

**Figure 5:**
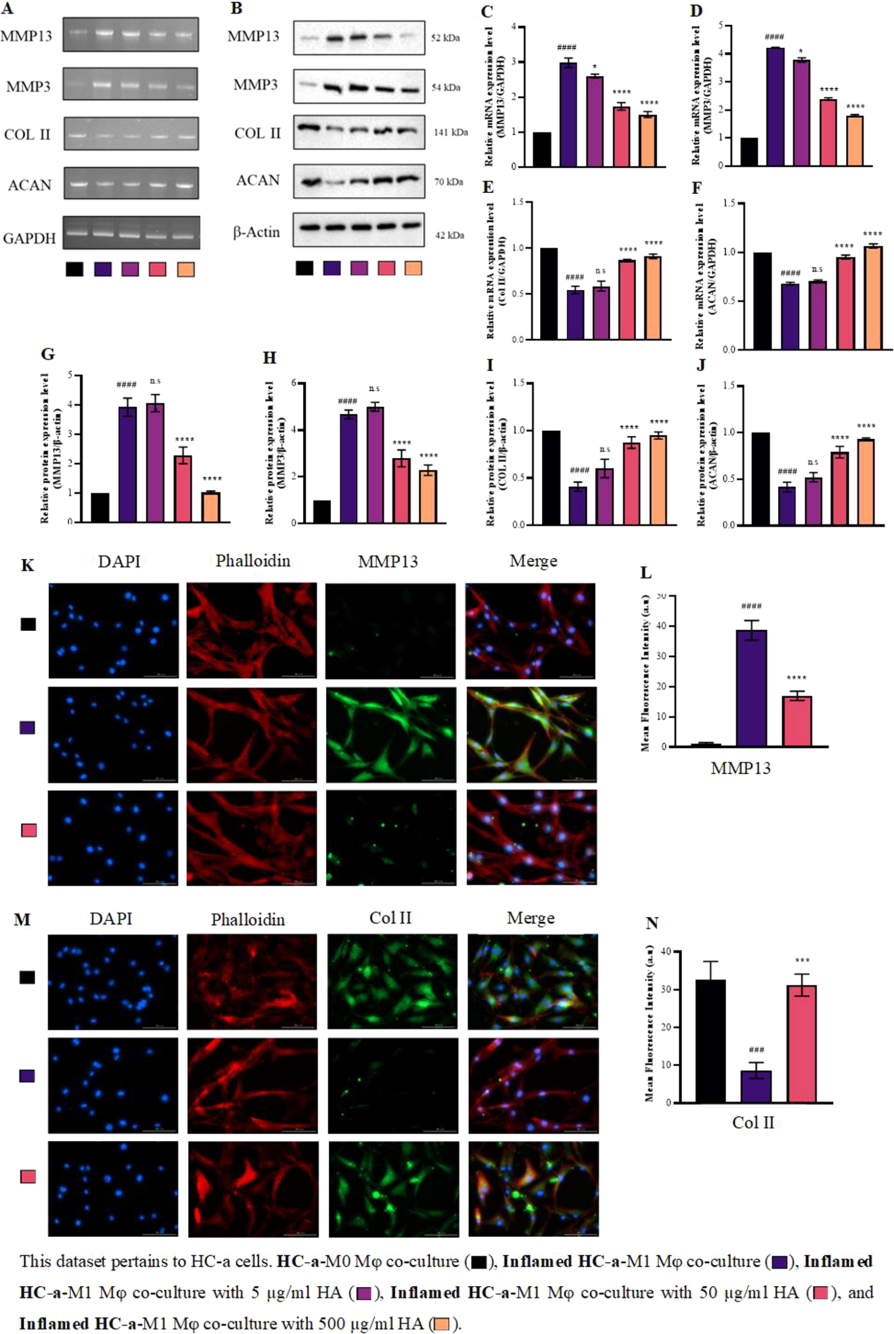
Effect of viscosupplement on gene expression, protein expression, and immunofluoresence of key inflammatory markers in HC-a cells cocultured with Mφ cells in the presence and absence of HA. (A) Gene expression and (B) protein expression patterns of MMP13, MMP3, Col II, and ACAN and the relative levels of (C–F) mRNA and (G–J) protein expression. Immunofluorescence staining of (K) MMP13 and (L) quantification of fluorescence intensity. Immunofluorescence staining of (M) Col II and (N) quantification of fluorescence intensity (bars = 100 μm; original magnification ×20). (### *p* < 0.001, #### *p* < 0.0001 compared to the control (HC-a-M0Mφ coculture group); n.s.: no significance; * *p* < 0.05, *** *p* < 0.001, and **** *p* < 0.0001 compared to inflamed HC-a (inflamed HC-a-M1Mφ coculture group).

In this study, we investigated the gene and protein expression patterns of HC-a within a coculture model. HC-a cells were cultured alongside THP-1-derived Mφ for 24 h and examined under different experimental conditions. The study consisted of three main groups. First, the coculture control group involved coculturing noninflamed HC-a and M0Mφ to observe baseline gene and protein expression patterns. Second, the inflamed coculture group involved the intentional inflaming of HC-a and M1Mφ before coculture to study their interactions and observe gene and protein expression changes over the 24 h period. Lastly, the inflamed coculture group was added with different concentrations (5, 50, and 500 μg/mL) of HA to investigate the potential impact of HA on gene and protein expression in inflamed HC-a and Mφ (Supplementary Figure 5 shows the cytotoxicity of HA on both cell types). The coculture control showed minimal changes in the expression patterns of the examined genes and proteins, indicating that the absence of inflammation had little impact on the gene and protein expression profiles of HC-a. By contrast, the inflamed coculture group, where inflamed HC-a and M1Mφ interacted for 24 h, exhibited a significant upregulation of proinflammatory markers in HC-a. Notably, HC-a cells (Figure 5A,B) showed increased expression level of MMP3 and MMP13, which are matrix-degrading enzymes associated with inflammation and cartilage degradation. At 50 and 500 μg/mL HA, a notable decrease was observed in the expression level of MMP3 and MMP13 in HC-a compared with the inflamed coculture without HA. In addition, we observed a significant upregulation of key genes associated with cartilage matrix synthesis, namely, Col II and ACAN, in HC-a treated with HA. Considering that 50 μg/mL showed significant results on par and sometimes equivalent to 500 μg/mL, we decided to narrow down the concentration to 50 μg/mL for further experiments. Immunofluorescent staining of MMP13 and Col II (Figure 5K,M) validated the protein expression patterns consistent with the previously described gene expression findings.

### Mechanistic Impact of HA on HC-a in Coculture with Mφ

**Figure 6:**
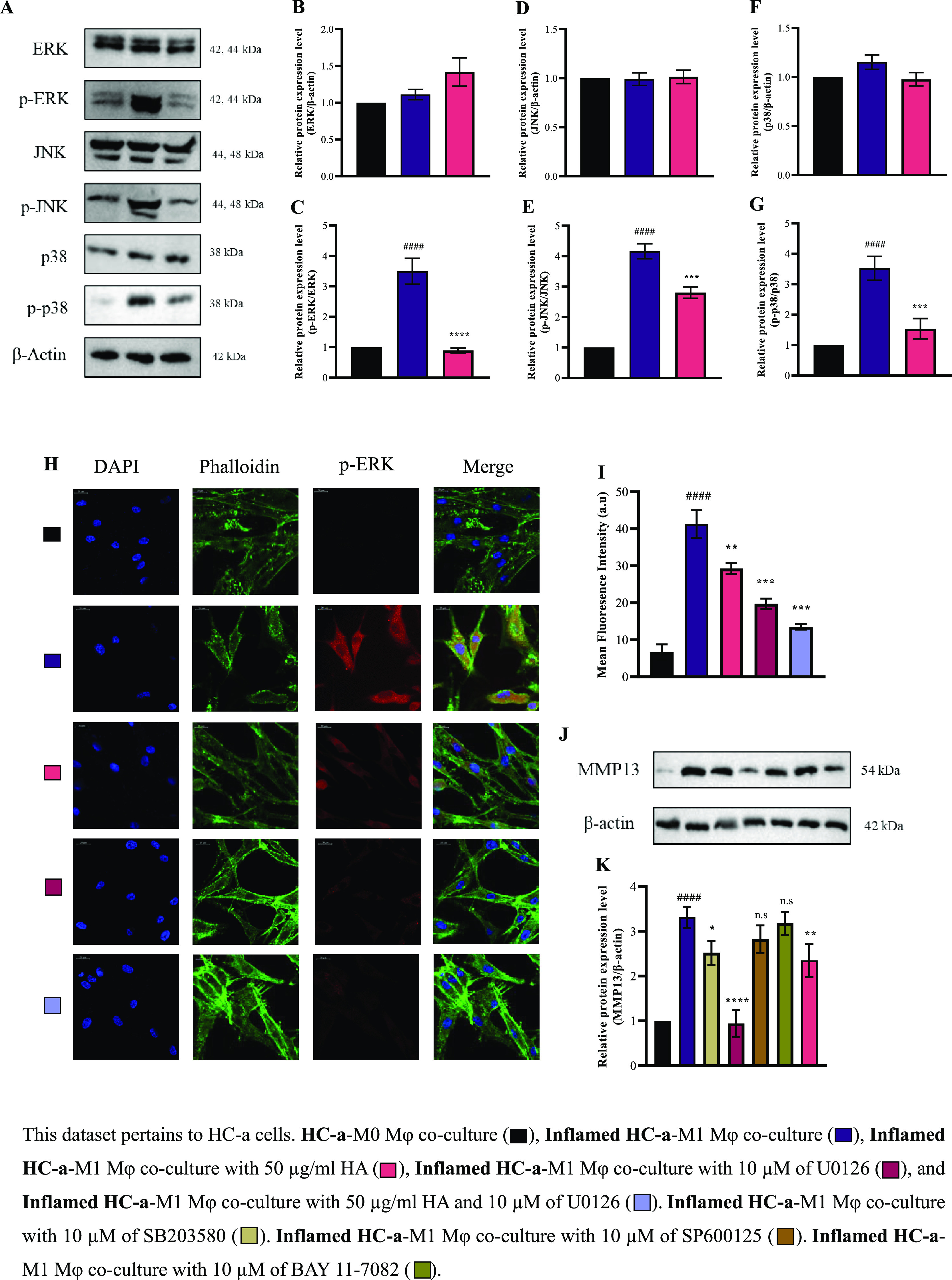
HA viscosupplement reduces inflammation via the MAPK pathway. (A) Protein expression and (B–G) quantitative analyses of total and phosphorylated forms of ERK, JNK, and p38 protein expression levels. (H) Confocal microscopy image of HC-a cells stained with pERK (red) antibody. Nuclei are counterstained with DAPI (blue) and (I) quantitative analysis. Scale bar represents 20 μm. Image was acquired using a 40× objective lens and processed using ImageJ software. Effect of HA on the expression of MMP13 protein in HC-a cells to determine the downstream effects of different signaling inhibitors (SB203580, U0126, SP600125 and BAY 11-7082) as evaluated through (J) protein expression levels and (K) quantitative analysis. (#### *p* < 0.0001 compared to the control group (HC-a-M0Mφ coculture group); n.s.: no significance; * *p* < 0.05, ** *p* < 0.01, *** *p* < 0.001, and **** *p* < 0.0001 compared to inflamed group (inflamed HC-a-M1Mφ coculture group).

We aimed to understand the mechanism by which HA reduces inflammation in HC-a cells, specifically by looking at its effects on decreasing the levels of proinflammatory mediators such as MMP13. After testing various HA concentrations, we found that 50 μg/mL resulted in a notable decrease in the levels of inflammatory mediators and higher levels of anti-inflammatory mediators. Thus, we chose this concentration for further research. In understanding the pathways involved in HA, we explored the MAPK pathways in HC-a cells (Figure 6A), observing a significant decrease in the expression level of the phosphorylated forms of pERK, pJNK, and pp38. Furthermore, we used specific inhibitors, such as U0126, SB203580, and SP600125, for MAPK to investigate the specific pathways through which HA reduces the mediators. The functional involvement of the protein pERK was substantiated via confocal microscopy, revealing apparent pERK inhibition by HA as evidenced by a reduced intensity in HC-a cells (Figure 6H). Remarkably, the addition of PERK-specific inhibitor U0126 resulted in significant reductions in the expression level of MMP13, highlighting its potential role in mediating the anti-inflammatory effects of HA on HC-a cells (Figure 6J).

### Suppression of Proinflammatory Cytokine Secretion in Inflamed Mφ through HA in a Coculture System

**Figure 7:**
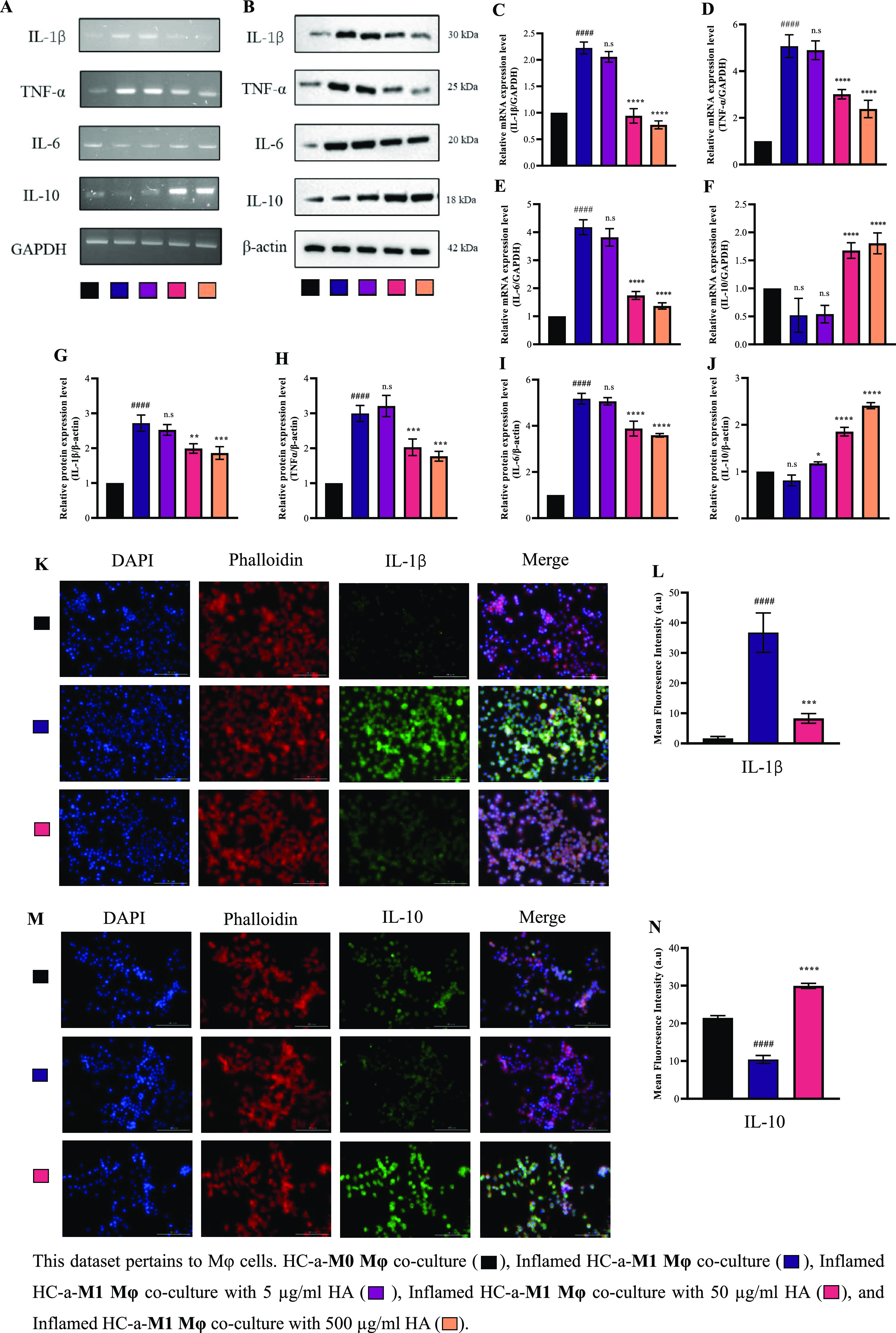
Effect of viscosupplement on gene expression, protein expression, and immunofluorescence of key inflammatory markers in Mφ cells cocultured with HC-a cells in the presence and absence of HA. (A) Gene and (B) protein expression patterns of IL-1β, TNF-α, IL-6, and IL-10 and (C–J) relative levels of mRNA and protein. Immunofluorescence staining of (K) IL-1β and (L) quantification of fluorescence intensity. Immunofluorescence staining of (M) IL-10 and (N) quantification of fluorescence intensity (bars = 100 μm; original magnification ×20). (### *p* < 0.001, #### *p* < 0.0001 compared to the control group (HC-a-M0Mφ coculture group); n.s.: no significance; * *p* < 0.05, ** *p* < 0.01, *** *p* < 0.001, and **** *p* < 0.0001 compared to inflamed group (inflamed HC-a-M1Mφ coculture group).

After investigating the mechanism by which the paracrine interaction affects HC-a cells, we observed the mechanism by which THP-1-derived Mφ reacts when placed in a coculture with HC-a. We examined the same coculture groups as we did when studying HC-a cells. In the inflamed coculture group, the levels of IL-1β, TNF-α, and IL-6—cytokines often associated with inflammation—were found to be increased in M1Mφ (Figure 7A,B). Notably, the addition of HA to the coculture environment had a significant suppressive effect on the inflammatory response of M1Mφ. In the presence of HA at 50 and 500 μg/mL, an apparent reduction in the production of proinflammatory cytokines (IL-1β, TNF-α, and IL-6) was observed in M1Mφ as compared with that in the inflamed coculture M1Mφ lacking HA. Moreover, the use of HA led to a significant increase in the anti-inflammatory mediator IL-10 within Mφ. Considering that 50 μg/mL showed significant results on par and sometimes equivalent to 500 μg/mL, we narrowed the concentration to 50 μg/mL for further experiments. Immunofluorescence analysis of IL-1β and IL-10 proteins confirmed their expression patterns, corroborating the observed gene expression results (Figure 7K,M).

### Mechanistic Impact of HA in Mφ Cocultured with HC-a

**Figure 8:**
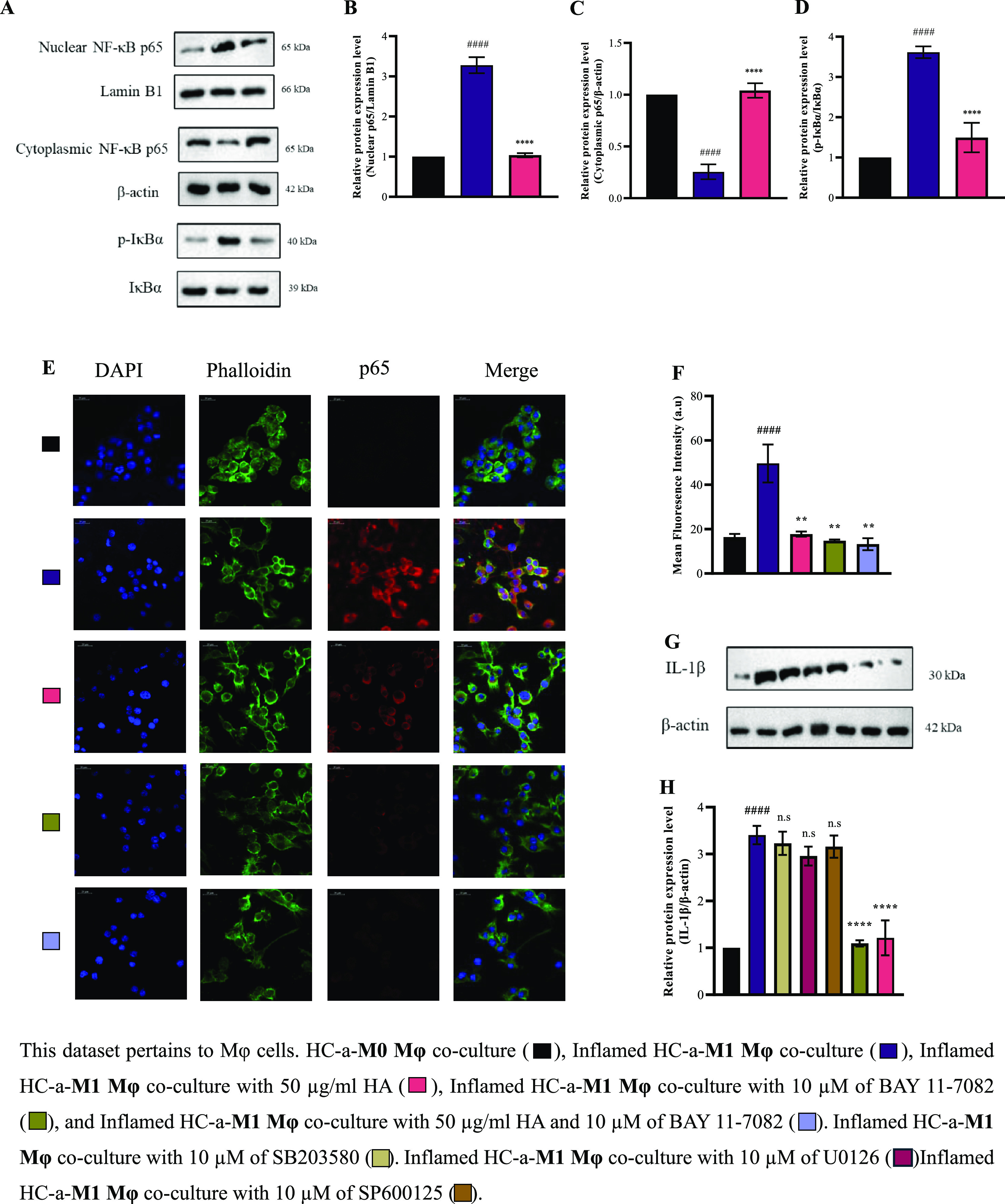
HA viscosupplement reduces inflammation via the NF-κB pathway in Mφ cells cocultured with HC-a. (A) Protein expression and (B–D) quantitative analyses of total and phosphorylated forms of IκBα and cytoplasmic and nuclear p65 protein expression levels. (E) Confocal microscopy image of Mφ cells stained with p65 (red) antibody. Nuclei are counterstained with DAPI (blue). Scale bar represents 20 μm. Image was acquired using a 40x objective lens. (F) Quantitative analysis of p65. Effect of HA on the expression of IL-1β protein in Mφ cells to determine the downstream effects of NF-κB inhibition with different signaling inhibitors (SB203580, U0126, SP600125, and BAY 11-7082) as evaluated through (G) protein expression levels and (H) quantitative analysis. (### *p* < 0.001, #### *p* < 0.0001 compared to the control group (HC-a-M0Mφ coculture group); n.s.: no significance; ** *p* < 0.01 and **** *p* < 0.0001 compared to inflamed group (inflamed HC-a-M1Mφ coculture group).

Next, we aimed to understand the underlying processes within Mφ, particularly on the role of NF-κB in inflammation, as previous studies have established its significance.^21,22^ This study explored whether 50 μg/mL HA could inhibit the nuclear translocation of NF-κB p65. As shown in Figure 8A, HA clearly inhibited the translocation of NF-κB p65 into the nucleus within THP-1-derived M1Mφ. This observation was reinforced through the use of confocal microscopy, which confirmed the attenuation of the NF-κB p65 translocation from the cytoplasm to the nucleus (Figure 8E). The results indicated that in M1Mφ, NF-κB p65 translocates into the nucleus. Pretreatment with BAY 11-7082 before LPS and IFN-γ stimulation effectively prevented NF-κB p65 from moving into the nucleus, thereby retaining it in the cytoplasm. HA showed outcomes analogous to those of BAY 11-7082 treatment. These outcomes indicated that HA curbed the presence of NF-κB in M1Mφ by mitigating its translocation, thereby reducing its impact. This study further investigated the involvement of NF-κB in the expression of IL-1β within activated M1Mφ. As depicted in Figure 8G, pretreatment of cells with 10 μM Bay11-7082 reduced the expression level of IL-1β, which is consistent with the western blot results. These findings highlighted the central role of NF-κB in modulating IL-1β, IL-6, and TNF-α levels in M1Mφ cocultured with HC-a cells.

## Discussion

OA poses a significant challenge despite the various treatment options and surgeries available to date. People with OA often experience persistent symptoms and side effects, raising concerns about the effectiveness of existing interventions. Although some medications have been proven to be effective in reducing inflammation and modifying cartilage mechanics, they fall short in fully restoring the essential qualities of synovial fluid. Addressing this gap, intra-articular viscosupplement injections have emerged as a favored alternative for maintaining lubrication and combating inflammation. Among these viscosupplements, HA is the most extensively used because of its well-established and compelling benefits in reducing inflammation.^23^ Animal and human studies over recent years have proven the efficacy and anti-inflammatory potential of HA.

In this study, we successfully established an in vitro coculture model that mimics OA conditions, facilitating the investigation of the Hyruan Plus function and mechanism within the joint. Although coculture models involving HC-a and Mφ have been explored, their interaction with viscosupplements such as Hyruan Plus remains largely unexplored despite their clinical relevance. Our focus was on the impact of viscosupplement on the paracrine interactions between HC-a and Mφ within the context of OA. In ensuring consistent conditions, we used chondrocyte and Mφ cell lines instead of cells from tissues with varying OA severity. Our results provide a reliable platform for exploring the effects and mechanisms of viscosupplements within the context of OA. From this study, M1Mφ cocultured with inflamed HC-a cells released proinflammatory cytokines and mediators compared with M0Mφ cocultured with noninflamed HC-a cells, contributing to OA pathogenesis. Similarly, a relevant study demonstrated that M1Mφ, triggered by LPS and IFN-γ, induced the expression of inflammatory markers, including IL-6, IL-1β, TNF-α, and iNOS. This finding highlights the potential ability of M1Mφ to release inflammatory mediators, potentially contributing to cartilage degradation in OA.^24^ Furthermore, we induced inflammation in HC-a with IL-1β, which was confirmed by the increased expression level of matrix-degrading enzymes (MMP3 and MMP13) and reduced expression level of cartilage matrix components (Col II and ACAN). In a coculture with M1Mφ, these effects were more pronounced, emphasizing the impact of inflammation on the matrix degradation.

Furthermore, when examining the impact of HA on inflammation in the coculture system, this study found that inflammation in Mφ triggered increased expression of proinflammatory markers in HC-a. This result highlights the interconnected relationship between Mφ and HC-a, indicating that inflammation in macrophages can initiate inflammation in chondrocytes, and emphasizes the intricate interplay among these cell types during inflammatory responses. In understanding this interaction, we initially observed the increased expression of proinflammatory markers in HC-a cells in the inflamed coculture group compared with that in the HC-a and M0Mφ coculture control group, emphasizing the interplay between Mφ and HC-a inflammation. This approach led us to examine the potential effects of HA on the interactions between HC-a and Mφ within the coculture system. After 24 h of coculture with or without the viscosupplement, we individually analyzed the gene and protein expression profiles of HC-a and Mφ. HA had a significant anti-inflammatory effect on HC-a and Mφ. In particular, treatment with the viscosupplement, especially at a concentration of 50 μg/mL, reduced the expression level of proinflammatory mediators (MMP13 and MMP3) in HC-a while increasing that of anti-inflammatory mediators (Col II and ACAN). This result indicates the potential of the viscosupplement to mitigate inflammation and promote cartilage matrix synthesis, contributing to joint health. Moreover, prior research reveals the capacity of HA to inhibit proinflammatory mediators in chondrocytes,^25^ thereby reducing MMPs, especially MMP1, 3, and 13, which play crucial roles in cartilage breakdown.^26^ Numerous reports have indicated that intra-articular HA has disease-modifying properties on cartilage, including the suppression of cartilage degeneration, enhanced chondrocyte density and morphology, and the prevention of cartilage surface fissures and cracks.^27−29^ In a recent study investigating the potential benefits of intra-articular Hyruan Plus injections in patients with early or intermediate-grade ankle OA who were unresponsive to conventional medications, three weekly injections of hyaluronate were not only safe but also effective in reducing pain and enhancing functional outcomes.^30^ This result further highlights the potential application of HA in aiding cartilage repair and maintenance.

In response to inflammation, Mφ within the coculture system upregulated the expression level of proinflammatory cytokines (IL-1β, TNF-α, and IL-6). However, the presence of HA had a suppressive effect on the Mφ inflammatory response, indicating its anti-inflammatory potential. Notably, HA treatment at a concentration of 50 μg/mL significantly reduced the production of proinflammatory cytokines and elevated the expression level of the anti-inflammatory cytokine IL-10 within Mφ. This result indicates the great impact of HA on modulating the immune response. Overall, the results demonstrated that HA treatment in an inflamed coculture setting can effectively mitigate the inflammatory response in Mφ. Mφ significantly affects gene expression related to cartilage matrix synthesis in chondrocytes and plays a pivotal role in the pathogenesis of OA by secreting key cytokines, such as IL-1β and TNF-α, which contribute to cartilage degradation.^31,32^ In our coculture model, we observed that HC-a displayed an increased expression level of matrix-degrading enzymes when cocultured, demonstrating the impact of Mφ on HC-a cells. Furthermore, the reduction of inflammatory markers in Mφ caused by HA treatment resulted in decreased inflammation in the HC-a cells. Therefore, Mφ-derived signaling negatively affects the critical components of HC-a cells, emphasizing the potential application of HA as an anti-inflammatory agent. These findings indicate the capacity of HA to promote cartilage repair, maintain immune homeostasis in the joint microenvironment, and modulate the Mφ activity.

**Figure 9:**
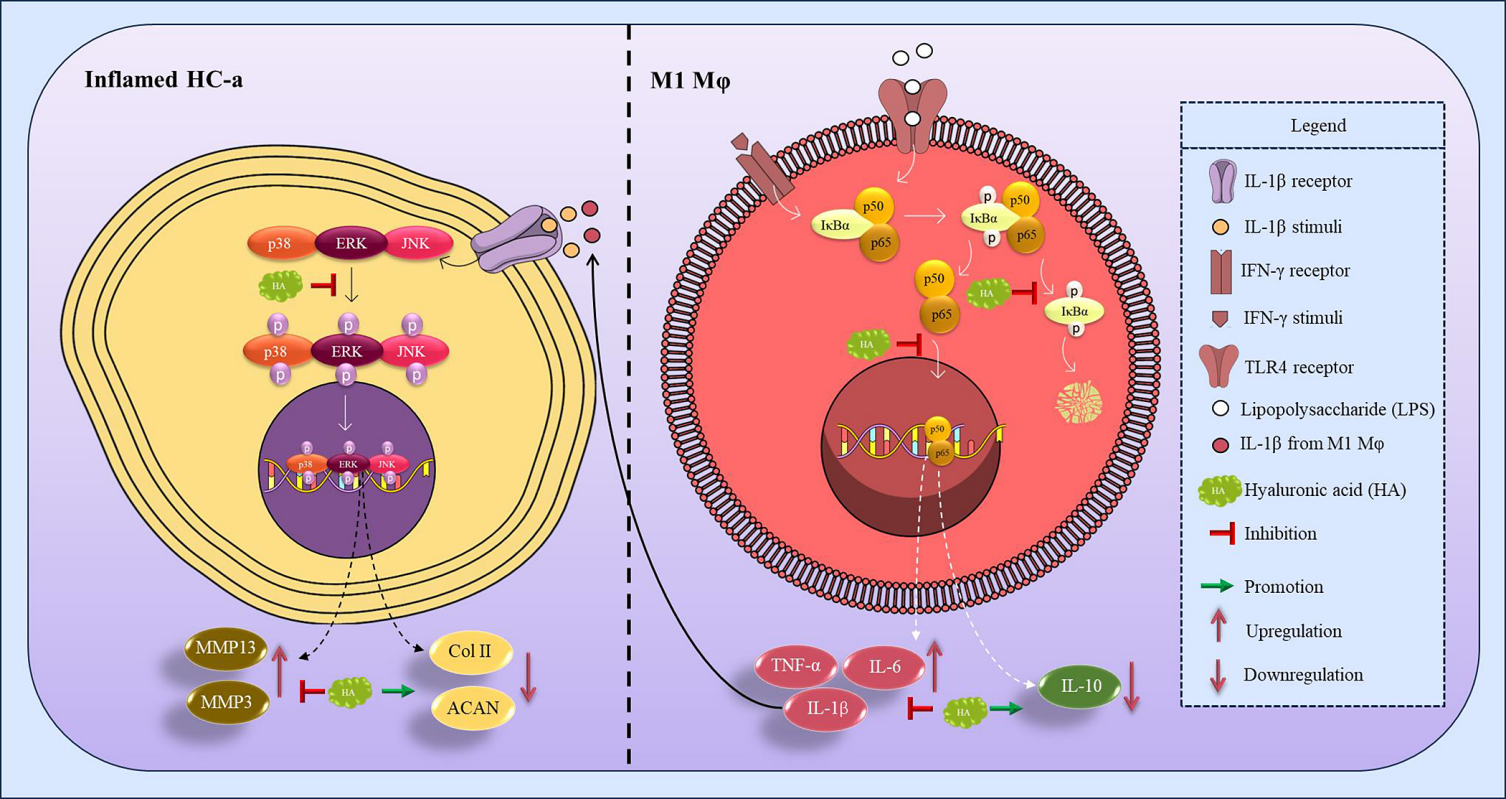
Proposed mechanism of HA in inflamed HC-a-M1Mφ paracrine interplay. This figure depicts the early inflammatory response elicited in inflamed HC-a and M1Mφ by IL-1β and LPS/IFN-γ, respectively. M1Mφ in coculture consistently induces inflamed HC-a cells to generate more matrix-degrading enzymes, hence sustaining the inflammatory cycle. The introduction of hyaluronic acid (HA) viscosupplement disrupts this cycle by inhibiting the phosphorylation of the MAPK and NF-κB pathways, therefore stopping the generation of inflammatory mediators and breaking the inflammatory contact loop between Mφ and HC-a cells.

In comprehensively investigating the pathways involved in our study, we focused on the MAPK pathway in HC-a cells and the NFκB pathway in Mφ (Figure 9). In our examination of HC-a cells, we noted a significant reduction in the expression levels of the phosphorylated forms of pERK, pJNK, and pp38. This decrease in phosphorylation prompted our hypothesis that the in vitro coculture model might also follow the same MAPK pathway, which has been demonstrated to play a crucial role in inflammation regulation in OA.^33,34^ Notably, all three phosphorylated forms, namely, pERK, pJNK, and pp38, exhibited a substantial decrease in the presence of 50 μg/mL of HA, but the ERK pathway played a central role in regulating MMP13 expression in HC-a cells cocultured with M1Mφ cells. (Supplementary Figure 6 shows a concentration-dependent decrease in the ERK signal observed in HC-a cells. These data support our finding that HA at a concentration of 50 μg/mL leads to a reduction in inflammation compared to other concentrations of HA ranging from 5 to 50 μg/mL.) In particular, the inhibition of pERK with U0126 demonstrated a key role in mediating the anti-inflammatory effects of HA by leading to substantial reductions in the MMP13 expression level. This observation is consistent with previous studies that demonstrated the activation of the ERK pathway in response to MMP13 induction in rabbit chondrocytes^35^ and the involvement of ERK in the regulation of MMP13 expression.^36^ In addition, HA inhibits the transcriptional activity of type α2(VI) collagen induced by IL-1β.^37^ HA can also prevent apoptosis induced by anti-Fas in human chondrocytes through its interaction with CD44 and CD54. Furthermore, HA reduces synovial hypertrophy, Mφ, lymphocytes, mast cells, and cartilage matrix degradation in OA.^38^ These mechanistic insights shed light on the mechanism by which HA exerts its anti-inflammatory benefits by influencing specific molecular pathways.

Our exploration of Mφ responses further revealed the critical involvement of the NF-κB pathway in inflammation, which is consistent with prior research. The NF-κB pathway plays a pivotal role in regulating the inflammatory response of Mφ, particularly in their polarization toward the M1 proinflammatory phenotype.^39^ This transition from M0 to M1, characterized by increased inflammatory properties, is significantly influenced by NF-κB signaling.^22^ Modulating this pathway inhibits the progression of M0Mφ toward the M1 phenotype, guiding them toward the anti-inflammatory M2 phenotype. We demonstrated that HA treatment at 50 μg/mL effectively suppressed the nuclear translocation of NF-κB p65 in M1Mφ, a response comparable to the known NF-κB inhibitor, BAY 11-7082. (Supplementary Figure 6 shows a concentration-dependent decrease in the NF-κB signal in Mφ cells. These data support our finding that HA at a concentration of 50 μg/mL leads to a reduction in inflammation compared to other concentrations of HA ranging from 5 to 50 μg/mL.) Our investigation demonstrated the significant involvement of the NF-κB pathway in regulating the expression level of proinflammatory cytokines (IL-1β, IL-6, and TNF-α) in M1 macrophages cocultured with HC-a cells, indicating the crucial role of NF-κB in the inflammatory response. In addition, we explored the contribution of IL-1β, a recognized NF-κB target gene, to OA progression. Existing research indicates that IL-1β binds to its receptor, activating the IκB kinase complex. This activation allows IκB phosphorylation, enabling NF-κB to enter the nucleus and release various inflammatory cytokines, including IL-1β.^40^ The IL-1β/NF-κB axis emerges as a potential therapeutic target for OA.^41^ Our findings revealed that the use of the NF-κB inhibitor BAY 11-7082 reduced the release of inflammatory cytokines by inhibiting IκB kinase phosphorylation. Moreover, HA exhibited a similar trend in suppressing the inflammatory cytokine production. Combining the NF-κB inhibitor with HA demonstrated that HA also operates within the NF-κB pathway, further inhibiting the IL-1β release. Furthermore, our study highlighted the pivotal role of the molecular weight of HA in reducing inflammation. The study by Lee et al. indicated that HMWHA mitigates synovial inflammation through the inhibition of the GRP78/NF-κB inflammatory pathway and proinflammatory cytokines, possibly by binding to ICAM-1.^42^ These findings are consistent with a systematic review that highlighted the molecular weight–dependent anti-inflammatory effects of HA through CD44, TLR, and ICAM receptor interactions. The molecular weight of HA was also found to be a significant factor, as increasing concentrations of HMWHA reduced NO production in LPS-stimulated Mφ.^43,44^ Our study further supports these findings by demonstrating the reduction of NF-κB expression and the suppression of proinflammatory cytokine release from Mφ, especially with a viscosupplement of 3000 kDa.

In this study, we also encounter some challenges. First, we used a coculture model, which, despite being helpful, does not mimic a real joint. The reduction in proinflammatory markers is due to the controlled environment that we created in our model. However, it is important to remember that the complexity of the natural joint with its different cell types must be considered. Moreover, it is essential to realize that viscosupplements, despite being effective, provide only temporary relief. They are typically an option for people who want to avoid surgery or cannot use steroid-based treatments because of allergies. In addition, a 24 h time frame was selected to study the immediate effects of the viscosupplement on the cells, but usually in humans, the typical duration of viscosupplementation treatment for OA of the knee is usually between 3 and 5 weeks based on pain relief in patients. The treatment involves injecting HA into the knee joint, with three to five injections at 1 week intervals. However, our primary objective was to establish an in vitro model that can mimic joint conditions to a certain extent, enabling a detailed exploration of the mechanisms underlying the action of viscosupplements as needed. Overall, our results provide a comprehensive understanding of the molecular mechanisms involved in OA. We have underscored the crucial role of proinflammatory cytokines, such as IL-1β and TNF-α, released by Mφ, and their downstream signaling pathways in promoting MMP expression and sustaining catabolic processes that contribute to joint tissue deterioration in OA. Moreover, we highlighted the potential of viscosupplement HA in improving inflammation by downregulating the MAPK and NF-κB signaling pathways, providing a promising avenue for therapeutic interventions in inflammatory joint diseases.

## Conclusions

This study successfully established an in vitro coculture model to investigate the function and mechanisms of viscosupplements, particularly HA, within the context of OA. Through the differentiation and activation of Mφ into the M1 inflammatory phenotype and the induction of inflammation in HC-a cells, we effectively simulated the inflammatory conditions that are characteristic of OA. The research revealed that HA treatment exerted significant anti-inflammatory effects on HC-a cells and Mφ by modulating key pathways such as MAPK and NF-κB. Notably, HA treatment demonstrated a remarkable ability to suppress the production of proinflammatory cytokines and enhance the expression level of anti-inflammatory markers in both cell types. These findings indicate that Hyruan Plus holds promising potential as a therapeutic agent for mitigating inflammation and promoting joint health in patients with OA. This study highlights the importance of understanding the molecular mechanisms underlying the anti-inflammatory properties of HA, paving the way for further investigations and potential clinical applications.

## Data Availability

All data generated or analyzed during this study are included in this article.

## Supporting Information Available

Morphological changes and gene expression in THP-1 cells differentiated in RPMI and DMEM culture media; effects of IFN-γ and LPS treatment on THP-1 Mφ viability; CD86 and CD206 immunofluorescence staining and IL-10 gene expression in M2Mφ; nitric oxide formation in cocultured HC-a and Mφ with varied HA concentrations; cell viability in HC-a and THP-1 Mφ with varied HA concentrations; and concentration-dependent effects of HA viscosupplement from 5 to 50 μg/mL on ERK in HC-a cells and NF-κB in Mφ cells (PDF)
