# Supplementary material for: Hyaluronic Acid Viscosupplement Modulates Inflammatory Mediators in Chondrocyte and Macrophage Coculture via MAPK and NF-κB Signaling Pathways
Source: ACS Omega. 2024 May 1;9(19):21467–83. doi: 10.1021/acsomega.4c01911 (PMC11097370; doi:10.1021/acsomega.4c01911)
Supplement: Supplementary file 1 — ao4c01911_si_001.pdf [file ao4c01911_si_001.pdf]

Hyaluronic Acid Viscosupplement Modulates Inflammatory Mediators in Chondrocyte and Macrophage Coculture via MAPK and NF-κB Signaling Pathways

Sree Samanvitha Kuppa[1,2,3,] Ju Yeon Kang[2,3,] Hong Yeol Yang[2,3,] Seok Cheol Lee[2,3,] Jaishree Sankaranarayanan[1,2,3,] Hyung Keun Kim[2,3*,] and Jong Keun Seon[1,2,3*]

[1]Department of Biomedical Sciences, Chonnam National University Medical School, Hwasun 58128, Korea

[2]Department of Orthopaedics Surgery, Center for Joint Disease of Chonnam National University Hwasun Hospital, 322 Seoyang-ro, Hwasun-eup, Jeonnam, 519-763, Korea

[3]Korea Biomedical Materials and Devices Innovation Research Center of Chonnam National University Hospital, 42, Jebong-ro, Dong-gu, Gwangju, 501-757, Korea

*Correspondence: Hyung Keun Kim (chemokines@naver.com) and Jong Keun Seon (seonbell@chonnam.ac.kr)

Table of Contents

Figure	Name	Page no.
S1	Morphological changes and gene expression in THP-1 Cells Differentiated in RPMI and DMEM Culture Media	3
S2	Effects of IFN-γ and LPS treatment on THP-1 Mφ viability	4
S3	CD86 and CD206 immunofluorescence staining and IL-10 gene expression in M2Mφ	5
S4	Nitric oxide formation in co-cultured HC-a and Mφ with varied HA concentrations	6
S5	Cell Viability in HC-a and THP-1 Mφ with varied HA concentrations	7
S6	Concentration-dependent effects of HA viscosupplement from 5- 50 μg/ml on ERK in HC-a cells and NF-κB in Mφ cells	8

Morphological changes and gene expression in THP-1 cells differentiated in RPMI and DMEM culture media

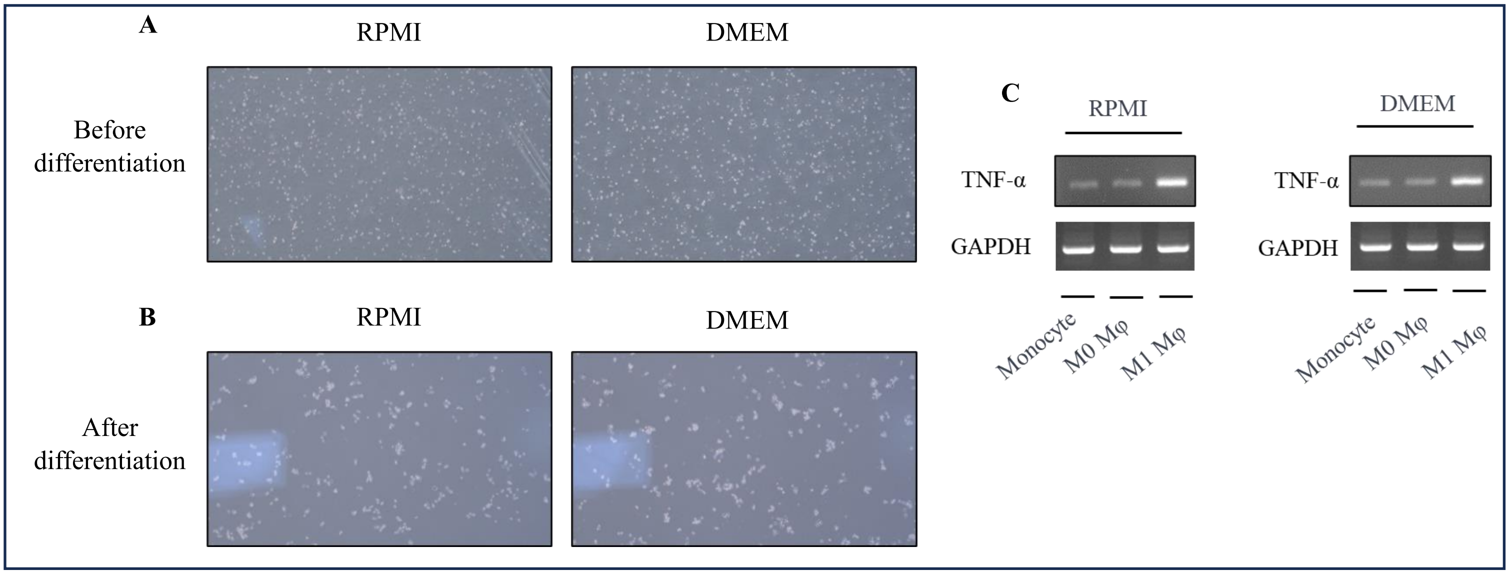

Supplementary Figure 1: Morphological characteristics of THP-1 cells were examined both (A) before and (B) after differentiation (treatment with 50 ng/ml PMA for 48 hrs) in RPMI and DMEM culture media. (C) Gene expression profiles of monocytes and Mφ (M0 and M1) were analyzed following cultivation in RPMI and DMEM culture media.

Effects of IFN-γ and LPS treatment on THP-1 Mφ viability

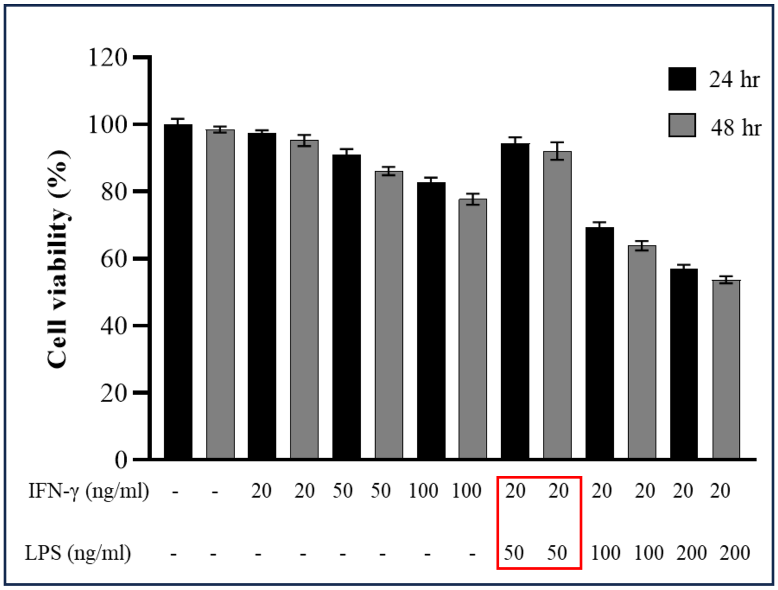

Supplementary Figure 2: THP-1 Mφs' were treated with IFN-γ (20, 50, or 100 ng/ml) and/or LPS (50, 100, or 200 ng/ml) and the viability of Mφ was investigated over 24 and 48 hours of incubation. Following the respective 24-hour and 48-hour incubation periods, the viability of the cells was assessed using the CCK-8 assay.

CD86 and CD206 immunofluorescence staining and IL-10 gene expression in M2 Mφ

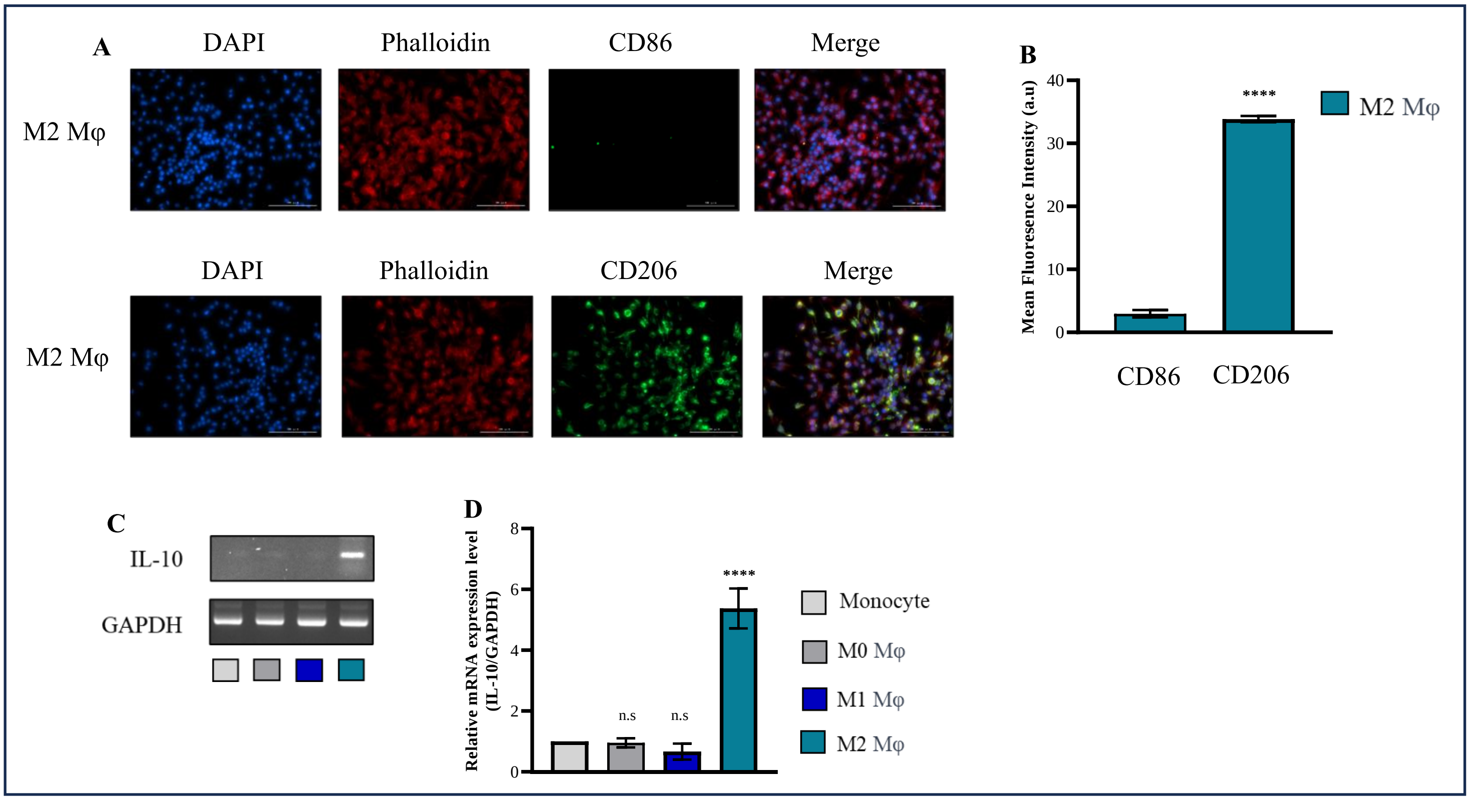

Supplementary Figure 3: The differentiation of M2Mφ followed a protocol similar to that of M1Mφ differentiation. Initially, 50 ng/ml of PMA was added to induce differentiation to M0Mφ. Following this differentiation step, 20 mg/ml of IL-4 was introduced for 24 h to differentiate the M0Mφ to M2Mφ. This polarization process was confirmed by assessing the immunofluorescence expression of surface markers, particularly CD206, a specific marker for M2Mφ, and CD86, which is typically absent in M2Mφ. (A) Immunofluorescence staining of CD86 and CD206 was performed on differentiated M2Mφ. (B) Qualitative analysis of CD86 and CD206 in M2Mφ. (C) Gene expression of IL-10 and (D) relative RNA expression level.

Nitric oxide formation in co-cultured HC-a and Mφ with varied HA concentrations

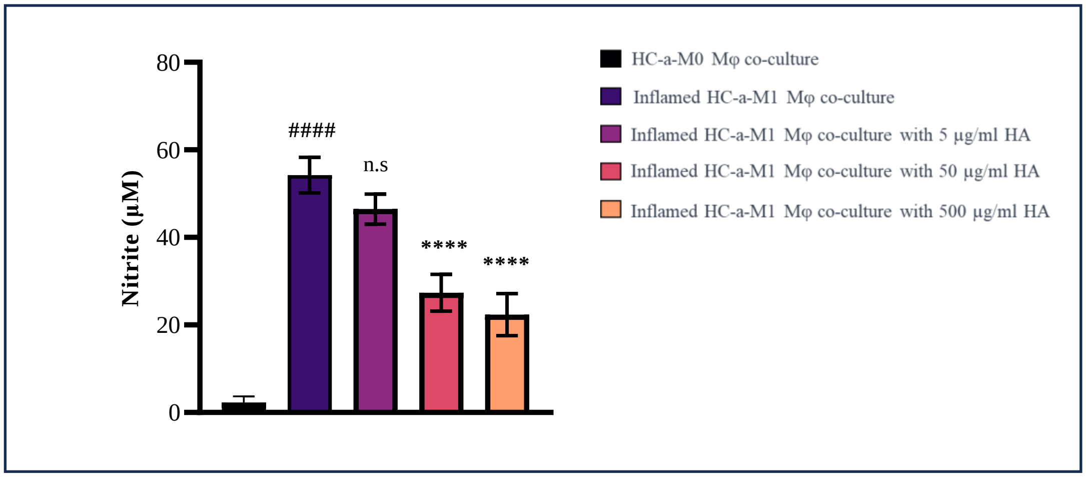

Supplementary Figure 4: HC-a and THP-1 derived Mφ were treated with varying concentrations of the HA viscosupplement (5, 50, or 500 µg/ml) for 24-hour and, the nitrite concentration of the cells was assessed using the Griess reagent. Statistical analysis revealed significant differences, denoted as ####$p < 0.0001$ compared to control (HC-a-M0Mφ co-culture group) and n.s (no significance) or ****$p < 0.0001$, when compared to Inflamed HC-a-M1Mφ co-culture group.

Cell Viability in HC-a and THP-1 Mφ with varied HA concentrations

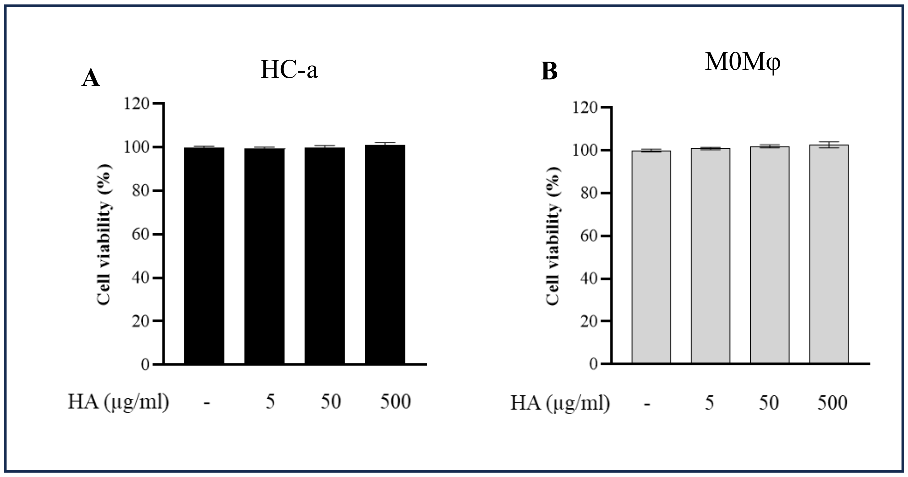

Supplementary Figure 5: The cell viability of (A) HC-a and (B) THP-1 M0Mφ was assessed with different concentrations of the HA viscosupplement (5, 50, or 500 μg/ml) for a duration of 24 hours. Subsequently, the cell viability was evaluated through the CCK-8 assay.

Concentration-dependent effects of HA viscosupplement from 5- 50 µg/ml on ERK in HC-a cells and NF-κB in Mφ cells

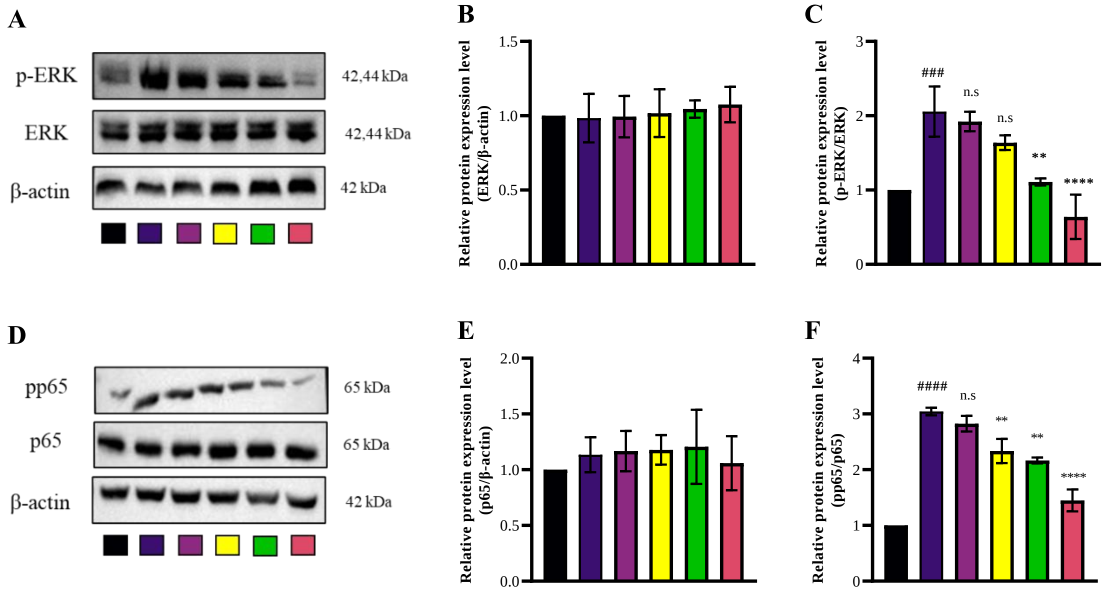

The (A-C) dataset pertains to HC-a cells and the dataset (D-F) pertains to Mφ. HC-a-M0 Mφ co-culture (■), Inflamed HC-a-M1 Mφ co-culture (■), Inflamed HC-a-M1 Mφ co-culture with 5 µg/ml HA (■), Inflamed HC-a-M1 Mφ co-culture with 10 µg/ml HA (■), Inflamed HC-a-M1 Mφ co-culture with 25 µg/ml HA (■), and Inflamed HC-a-M1 Mφ co-culture with 500 µg/ml HA (■).

Supplementary Figure 6: Concentration-dependent alterations in (A) ERK signaling in HC-a cells and (B-C) quantitative analyses of total and phosphorylated ERK protein expression. Similarly, concentration-dependent variations in (D) NF-κB (p65) signaling in Mφ cells and (E-F) quantitative analyses of total and phosphorylated p65 protein expression were evaluated using different concentrations of HA viscosupplement (5, 10, 25, and 50 µg/ml) via western blot analysis.
